# Context-Dependent Effects of *Trichoderma* Seed Inoculation on Anthracnose Disease and Seed Yield of Bean (*Phaseolus* *vulgaris*): Ambient Conditions Override Cultivar-Specific Differences

**DOI:** 10.3390/plants10081739

**Published:** 2021-08-23

**Authors:** Karina Gutiérrez-Moreno, Michelina Ruocco, Maurilia Maria Monti, Octavio Martínez de la Vega, Martin Heil

**Affiliations:** 1 Laboratorio de Ecología de Plantas, Departamento de Ingeniería Genética, Centro de Investigación y de Estudios Avanzados (CINVESTAV)—Unidad Irapuato, 36824 Irapuato, Mexico; karina.gutierrez@cinvestav.mx; 2 Institute for Sustainable Plant Protection, National Research Council (CNR-IPSP), Via Università 133, 80055 Portici, Italy; maurilia.monti@ipsp.cnr.it; 3 Laboratorio Nacional de Genómica para la Biodiversidad, Centro de Investigación y de Estudios Avanzados (CINVESTAV)—Unidad de Genómica Avanzada, 36824 Irapuato, Mexico; octavio.martinez@cinvestav.mx

**Keywords:** biocontrol, common bean, dry bean, fungus-plant interaction, plant disease, *Trichoderma atroviride* P1, *Trichoderma asperellum* B6, *Trichoderma longibrachiatum* MK1, *Trichoderma harzianum* T22

## Abstract

Root colonizing *Trichoderma* fungi can stimulate plant immunity, but net effects are strain × cultivar-specific and changing ambient conditions further contribute to variable outcomes. Here, we used four *Trichoderma* spp. to inoculate seeds of four common bean (*Phaseolus vulgaris*) cultivars and explored in three different experimental setups the effects on fungal anthracnose after leaf inoculation with *Colletotrichum lindemuthianum*. Plants growing in pots with field soil under greenhouse conditions exhibited the highest and those in the open field the lowest overall levels of disease. Among 48 *Trichoderma* strain × bean cultivar × setup combinations, *Trichoderma*-inoculation enhanced disease in six and decreased disease in ten cases, but with the exception of *T. asperellum* B6-inoculated Negro San Luis beans, the strain × cultivar-specific effects on anthracnose severity differed among the setups, and anthracnose severity did not predict seed yield in the open field. In the case of Flor de Mayo beans, *Trichoderma* even reduced yield in anthracnose-free field plots, although this effect was counterbalanced in anthracnose-infected plots. We consider our work as a case study that calls for stronger emphasis on field experiments in the early phases of screenings of *Trichoderma* inoculants as plant biostimulants.

## 1. Introduction

Plant roots are colonized by highly diverse microbiomes that comprise mutualistic, commensal and pathogenic bacteria and fungi [1]. Among the beneficial microorganisms, soilborne fungi in the genus *Trichoderma* stand out because they promote plant growth and benefit plant health via a particularly wide range of mechanisms, including the solubilization of nutrients, the protection from soilborne pathogens via direct antibiosis and mycoparasitism, and an activation of the plant’s innate immune system. All these mechanisms and effects have been reviewed, and reviewed well, by others [2,3,4,5,6,7,8,9,10]. A biological resistance induction that overcomes growth-resistance trade-offs has obvious potential for applications in biocontrol and therefore, intensive screening efforts are undertaken to find efficient *Trichoderma* strains for application to diverse agri- and horticultural crops as ‘biostimulants’ *sensu* du Jardín [11], ‘biopesticides’, ‘biofungicides’ or ‘growth enhancers’ [12,13,14,15].

However, the effects of *Trichoderma* spp. on plants can range from the promotion to the inhibition of growth and from inducing plant resistance to enhancing their host´s susceptibility [16,17,18,19]. *Trichoderma*-plant interactions comprise complex molecular dialogues that trigger large-scale transcriptomic changes in both partners [4,6,7,20,21,22,23,24,25,26], and therefore, the fungal strain and plant genotype (or cultivar) are major determinants of the net outcome [24,25,27]. Soil type and fertilizer regime have been identified as additional factors that contribute to variation in, e.g., the effects of a specific *Trichoderma* strain on the growth, nutrient uptake or yield, of crops such as shallot (*Allium cepa*), tuberose (*Polianthes tuberosa*), tomato (*Lycopsersicum esculentum*), broccoli (*Brassica rapa* subsp. *sylvestris*) mustard (*Brassica rapa* L.), maize (*Zea mays*) or wheat (*Triticum aestivum*) [28,29,30,31,32,33]. Even under completely controlled conditions, a specific *T. asperellum* strain promoted the growth of *Arabidopsis thaliana* seedlings on MS medium, but had inhibitory effects in sterile soil [34]. Considering that both partners engage in this interaction with their own ‘goals’, the dependency on soil parameters and nutrient availability should not come as a surprise: from the perspective of the plant, *Trichoderma* fungi are only one element of a complex root microbiome whose relevance for plant health and nutrition depends on functions fulfilled by other members of this microbiome and nutrient availability, and also for *Trichoderma*, root exudates are only one out of various potential sources of nutrients [35,36,37]. Since any experimental intervention adds a new level of complexity to these interactions, even the seemingly simple question how to define the right control becomes difficult for experiments that include *Trichoderma* or other biocontrol organisms [38].

In summary, selecting *Trichoderma* strains for application to a specific crop requires screenings of multiple strain × cultivar combinations, and choosing the best experimental setup for this purpose remains a complicated task. Two- or three-partner systems kept under sterile conditions are not likely to reveal the full range of possible outcomes in the open field, while field studies are work-intensive and notoriously suffer from a low degree of reproducibility, because many environmental factors cannot be controlled [2]. Therefore, a common choice is to cultivate the plants under controlled ambient conditions, i.e., in growth chambers or greenhouses, in pots filled with sterile substrate, sterilized field soil or non-sterilized field soil. For example, non-sterilized potting compost or soil was used to study the effects of soil inoculation with various *Trichoderma* strains on the germination rate and early growth of lettuce (*Lactuca sativa*) [18], or of leaf inoculation with two *Trichoderma* and a *Streptomyces* strains on *Curvularia oryzae*-caused leaf spot disease of oil palm [39]. By contrast, sterile commercial substrates like ‘Sunshine Mix 3’ or composted ‘pine bark growth medium’ were used to study, e.g., effects of the inoculation with *T. harzianum* or *T. virens* on the growth of lentil (*Lens culinaris*) seedlings [16] or of *T. harzianum* or *T. atroviride* on nutrient uptake, growth rate and nodulation of common bean (*Phaseolus vulgaris*) seedlings [40]. However, soil matters even in such reductionistic setups: the effects of two *T.* *harzianum* strains on the growth, nutrient uptake and glucosinolate content of broccoli varied strongly among plants grown in pots in field soil collected from different sites [30], and seed inoculation with *Trichoderma saturnisporum* improved the seedling growth of tomato (*Solanum lycopersicum*), pepper (*Capsicum annuum*) and cucumber (*Cucumis sativus*) in a substrate rich in organic matter, but had negative effects in mineral soil [41]. Moreover, time in the greenhouse can be costly, for which reason most studies are limited to the quantification of seedling growth, which is not necessarily a reliable indicator of net seed yield.

Therefore, the aim of the present study was to explore the degree to which a potential *Trichoderma*-mediated systemic effect on disease resistance of common bean depends on ambient conditions. In order to use experimental setups that are commonly used for screening purposes, we inoculated bean seeds from four cultivars with each of four *Trichoderma* spp. (using not *Trichoderma*-inoculated seeds as controls) and cultivated the plants (A) in pots with a sterile, commercial substrate in a greenhouse, (B) in pots with non-sterile field soil in the greenhouse and (C) in open field plots. Considering that the type of soil, and in particular the content of organic matter and the presence of other microorganisms, has been identified as an important environmental determinant of the effects of a specific *Trichoderma* strain on the growth or yield of other crops [18,28,29,30,31,32,33,41,42], we hypothesized that plants growing in natural soil—but under otherwise controlled conditions (i.e., a greenhouse)—might be an attractive setup to select *Trichoderma* strains as potential biocontrol agents for bean.

We chose common bean (also known as dry bean or French bean) as a highly important staple crop and primary source of protein for humans in many developing countries [43,44,45,46] for which relatively few studies explored *Trichoderma* spp. as means of biological control [47], perhaps because a study that screened 101 strains of *Trichoderma* identified only seven strains with a growth promoting effect [48], or simply because bean generates limited economic gains. Besides the beforementioned screening, dosage-dependent effects of a *T. harzianum* inoculum on the nutrient uptake and seed yield of bean plants were reported from two studies under pathogen-free conditions [40,49]. In addition, *Trichoderma* spp. can trigger extensive metabolomic changes in bean plants [50] and induce the expression of pathogenesis-related proteins [51] or the activity of defense-related enzymes such as peroxidase, polyphenol oxidase and phenylalanine ammonia lyase [52]. Moreover, various strains of *T. asperellum* or *T. harzianum* have been shown to directly inhibit soilborne fungi that cause root rot and damping-off disease of *P. vulgaris*, thereby enhancing seedling emergence and early growth rate in soils contaminated with *Rhizoctonia solani* and *Fusarium solani*, or from *Phytium ultimum*-infected bean seeds [53,54,55,56,57]. While evidence for *Trichoderma*-mediated protection from soilborne disease by direct antibiosis is accumulating, few studies focused on systemic resistance, although *T. harzianum* seed inoculation has been reported to generate induced systemic resistance against *Uromyces appendiculatus,* the causal agent of bean rust [58].

Fungal anthracnose caused by *Colletotrichum lindemuthianum* is a further and particularly devastating disease of *P. vulgaris* that can lead to complete yield loss or render seeds unsuitable for consumption or commercialization [59,60,61,62]. The fungus is frequently transmitted via contaminated seeds or soil, and a pioneering study reported already in 1995 that several *Trichoderma* isolates can directly inhibit the growth of *C. lindemuthianum* [63]. However, *C. lindemuthianum* can also enter via the leaves and—independently of the original site of entry—establishes a systemic infection, while seed inoculation with *Trichoderma* usually results in little or no colonization of the aerial parts of a plant [58,64]. To the best of our knowledge, only one study reported a delay of anthracnose symptoms in the leaves of *P. vulgaris* after seed inoculation with *T. viride* or *T. tomentosum* and thereby shows that—in principle—*Trichoderma* spp. can trigger systemic resistance to *C. lindemuthianum* [65].

For the present study, we tested four *Trichoderma* strains from different species (*T. asperellum* B6, *T. longibrachiatum* MK1, *T. atroviride* P1 and *T. harzianum* T22) for their potential to reduce the symptoms of anthracnose in four *P. vulgaris* genotypes that cover a broad range of basal resistance to *C. lindemuthianum*; the cultivars Flor de Mayo Anita (FMA) and Pinto Villa (PV) are considered as ‘resistant’ [66,67], the cultivar Flor de Junio Marcela (FJM) has an intermediate level of resistance [68], while the landrace, Negro San Luis (NSL), is highly susceptible to *C. lindemuthianum* [69]. After challenging the leaves with *C. lindemuthianum*, *Trichoderma*-inoculated plants exhibited both increased and decreased disease levels as compared to *Trichoderma*-free controls and intriguingly, *Trichoderma* strain and experimental setup explained a larger degree of this variation than the cultivar of bean.

## 2. Results and Discussion

### 2.1. Ambient Conditions Override Cultivar-Specific Effects of Trichoderma-Inoculation on Anthracnose Severity of Bean Plants

**Figure 1:**
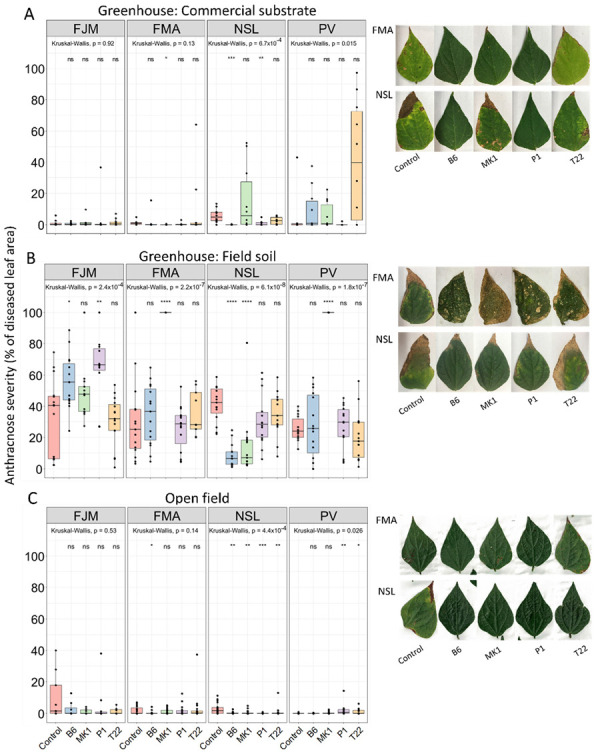
Effects of *Trichoderma* inoculation on anthracnose severity of bean plants cultivated in different experimental setups, (**A**) in pots with sterile substrate in a greenhouse, (**B**) in pots with non-sterile field soil in a greenhouse, (**C**) in the open field. Bean cultivars are, FJM: Flor de Junio Marcela; FMA: Flor de Mayo Anita; NSL: Negro San Luis; PV: Pinto Villa. Treatments, control: no *Trichoderma*; B6: *T. asperellum* B6; MK1: *T. longibrachiatum* MK1; P1: *T. atroviride* P1; T22: *T. harzianum* T22. Boxplots indicate medians, 25th and 75th percentiles, whiskers extend to 1.5 times the interquartile range, and data points beyond whiskers represent outliers. Asterisks indicate significant effects of *Trichoderma* inoculation on the anthracnose severity of plants of the same cultivar and in the same experimental setup (**** *p* ≤ 0.0001, *** *p* ≤ 0.001, ** *p* ≤ 0.01, * *p* ≤ 0.05, ns: *p* > 0.05; Wilcoxon post hoc tests, *n* = 10 independent biological replicates). Photos show representative examples of disease symptoms.

In the present study, we used four *Trichoderma* strains (plus mock-inoculation as *Trichoderma*-free control) to seed-inoculate four cultivars of common bean and cultivated the plants in three different setups (in a full factorial design of 5 × 4 × 3 = 60 strain × cultivar × setup combinations) to analyze the levels of anthracnose disease 20 days post-inoculation (dpi) of *C. lindemuthianum* to the leaves. We aimed to use, as response variable, a fitness-relevant phenotypic effect that is fast and easy to observe without costly equipment. Therefore, we quantified disease severity as percent of leaf area with visible symptoms. Plants cultivated in the greenhouse in field soil suffered from the highest disease severity, with diseased areas reaching on average 30–50% and in several cases 100% of the total leaf area. Plants cultivated in the greenhouse in sterile commercial substrate exhibited intermediate levels, and plants in the open field the lowest levels of disease, not passing 5% leaf area affected for any of the strain × cultivar combinations. Besides the different absolute levels of disease symptoms, we also observed considerable variation among the effects of *Trichoderma* on anthracnose severity. We analyzed the effects of seed inoculation with *Trichoderma* for each cultivar and setup using individual Wilcoxon post-hoc tests and discovered that the only strain × cultivar combination that exhibited a significant reduction in anthracnose disease as compared to mock-inoculated control in all three setups was *T. asperellum* B6-inoculated NSL beans. Among all strain × cultivar combinations, *Trichoderma*-inoculation was associated with significantly lower disease symptoms in ten cases and with significantly enhanced symptoms in six combinations (Figure 1).

Multivariate analysis of variance (MANOVA) of the full factorial design (with *n* = 10 biologically independent replicates per strain × cultivar × setup combination) confirmed significant effects (*p* < 0.05) for the *Trichoderma* treatment and the experimental setup, but not for the cultivar of bean (Table 1).

We detected highly significant interaction effects among the three factors (*p* < 0.001), a result indicating that the plant cultivar also contributed to the overall patterns in anthracnose disease levels (Table 1). Since the data did not fulfil the criterium of normality distribution (Shapiro-Wilk test, Supplementary Material Figure S1)—most likely due to the high abundance of low and very low values—we used non-parametric (Kruskal-Wallis) tests as an alternative statistical analysis and detected significant effects (*p* < 0.05) of all three factors (Table S1). Nevertheless, the exact *p*-values confirm that *Trichoderma* strain and experimental setup contributed most to the variation in the degree of anthracnose disease. While a protection from soilborne disease agents by *Trichoderma* can result mainly from direct antibiosis and mycoparasitism, i.e., plant-independent mechanisms, variation in the systemic effects of *Trichoderma* is typically observed in studies that compare different cultivars of the same plant species, e.g., maize and tomato colonized by *T. harzianum* T22 and *T. atroviride* P1 [25,64]. For tomato, a recent study reported a striking loss of the capacity to respond to *Trichoderma* with an induced systemic resistance to pathogens over the course of domestication [70]. The bean cultivars used in the present study are characterized by different levels of basal resistance to *Colletotrichum* [66,67,68,69], they also differ in their basal and inducible resistance to bacterial pathogens [71], and we inoculated *Colletotrichum* via the leaves of 3–5 wk-old plants. As argued by Abeysinghe for leaf rust [58], the resulting spatial separation excludes direct antibiosis as a potential mechanism by which seed-inoculated *Trichoderma* spp. could control anthracnose disease in the leaves and reproductive parts of bean plants. Thus, any *Trichoderma*-related effects on anthracnose levels should result mainly (if not completely) from plant-mediated mechanisms. In this sense, the relatively low contribution of cultivar-driven effects in our study is surprising.

Considering the importance of the soil type reported by others, we performed a separate analysis of the data obtained under greenhouse conditions (Figure 1A,B), which revealed significant effects (*p* < 0.05 according to Kruskal-Wallis tests, see Table S2) for fungal strain, bean cultivar and soil type (sterile substrate vs. field soil). Moreover, similarly to the complete dataset, the absolute values of the test variables (χ-square and *p*-values) confirm the type of growing substrate as a major determinant of the degrees of anthracnose, even if plants are cultivated in a greenhouse, i.e., under otherwise standardized ambient conditions.

### 2.2. Mortality and Damage by Non-Controlled Environmental Factors in the Field

Under open field conditions, many additional biotic and abiotic factors determine the net effects of *Trichoderma* spp. on plant growth, disease resistance and—ultimately—yield [2]. For example, inducing resistance to a specific type of stress can reduce the resistance of the plant to other factors [72,73,74]. Although *Trichoderma* spp. can trigger both salicylic acid- and jasmonic acid-dependent signaling [15,75], shifts from one to the other pathway have been reported, at least in tomato [76,77], and inoculating maize with *T. harzianum* caused increased abundances of chewing herbivores [78]. To study the effects of *Trichoderma* on bean plants under open field conditions, we used an experimental field that had been devoted to experimental anthracnose infections in earlier years. We established four plots in a split-plot design (Figure S2), each plot comprised all *Trichoderma* × cultivar-combinations (*n* = 8 seeds per strain × cultivar combination), seeds were sown directly into the soil in a completely randomized spatial distribution, and plants in the two ‘anthracnose-plots’ were leaf-challenged with *C. lindemuthianum*, while plants on the ‘anthracnose-free’ control plots were mock-challenged.

Since we did not apply any type of pesticide in our field plot, we decided to document plant mortality and damage by non-controlled biological enemies in form of three ‘snapshots’. Seed inoculation of common bean with several *T. harzianum* isolates had positive effects on seedling emergence in a field experiment performed in Brazil [79]. Therefore, we determined soilborne mortality as not emerged plantlets (of 8 seeds sown per strain × cultivar combination and subplot). In comparison with not *Trichoderma*-inoculated controls, *Trichoderma*-inoculated PV beans seemed to suffer from enhanced soilborne mortality on both subplots and *Trichoderma*-inoculated NSL beans showed the same effect on the anthracnose-plot (Table 2). Several *T. harzianum* and *T. longibrachiatum* isolates have been reported to increase post-emergence damping off in cotton seedlings [80]. However, we are not aware of a similar report for bean and therefore, can only speculate that *C. lindemuthianum*-conidia that remained in the soil from treatments in previous years have contributed to this effect.

**Figure 2:**
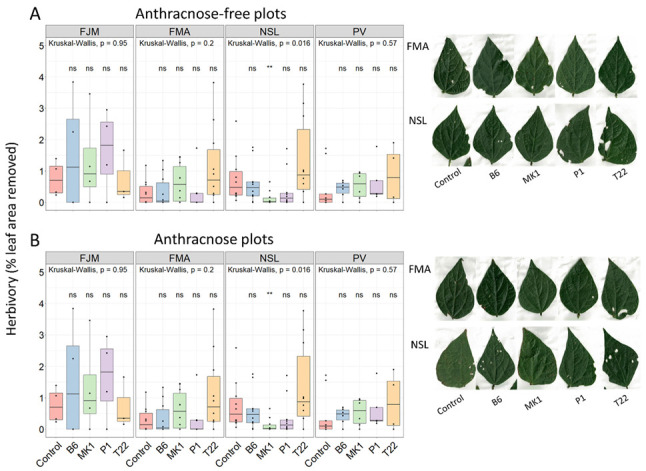
Herbivory levels of bean plants under open field conditions on (**A**) Anthracnose-free plots and (**B**) Anthracnose plots. Damage levels are expressed as percent missing leaf area and were quantified 20 d after leaf challenge with *C. lindemuthianum*. Common bean cultivars: FJM: Flor de Junio Marcela; FMA: Flor de Mayo Anita; NSL: Negro San Luis; PV: Pinto Villa. *Trichoderma*-treatments, Control: No *Trichoderma*; B6: *T. asperellum* B6; MK1: *T. longibrachiatum* MK1; P1: *T. atroviride* P1; T22: *T. harzianum* T22. Boxplots indicate medians, 25th and 75th percentiles, whiskers extend to 1.5 times the interquartile range, data points beyond whiskers represent outliers. Asterisks indicate significant effects of *Trichoderma* inoculation on herbivory among plants of the same cultivar (** *p* ≤ 0.01, * *p* ≤ 0.05, ns: *p* > 0.05; based on Wilcoxon post hoc tests of *n* = max. 10 independent biological replicates (or less in cases of high soilborne mortality). Photos show representative herbivore-inflicted damage.

We used the images taken at 20 dpi for the quantification of disease symptoms to determine the degree of herbivory. Overall, the plants suffered from relatively low levels of disease as well as herbivory (Figure S3). Nevertheless, we detected a significant effect of *C. lindemuthianum*-inoculation on the levels of herbivory (*p* < 0.01, see Table S3) but not for *Trichoderma* strain or bean cultivar (*p* > 0.05, Kruskal Wallis tests), although the exact *p*-values indicate ‘marginally significant’ effects (*p* = 0.053 for ‘cultivar’ and *p* = 0.057 for ‘*Trichoderma*’). More importantly, we detected a significant effect of *Trichoderma*-inoculation on herbivory only for three out of 32 strain × cultivar × anthracnose—combinations: in the anthracnose-free plot, MK1- and T22-inoculated PV beans suffered from significantly more damage by herbivores than non-inoculated plants, while MK1-inoclated NSL-beans suffered significantly less from herbivory in the anthracnose-plot (Figure 2).

Although the lack of significant within-cultivar effects indicates that seed inoculation with *Trichoderma* had a minor effect on herbivory, we conclude from the significant overall effect of *C. lindemuthianum*-inoculation on herbivory rates and visual inspection of the plants (Figure S4), that chewing herbivores were likely a major cause of plant mortality before reaching maturity (Table 2). In fact, the numbers of PV plants lost on the anthracnose-free plot indicate a potential positive effect of *Trichoderma* while curiously, much less *Trichoderma*-inoculated NSL plants than *Trichoderma*-free controls survived on the anthracnose-plots (Table 2). Although the effective sample size of *n* = 2 does not permit any robust statistical analysis, we consider these patterns as an observation that calls for further studies.

### 2.3. Seed Yield

**Figure 3:**
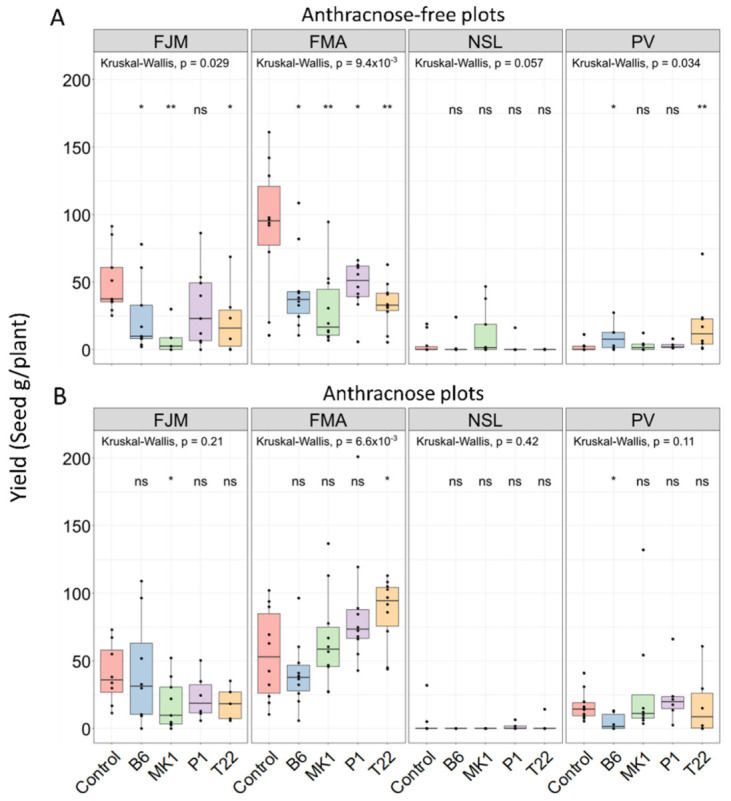
Effects of seed inoculation with *Trichoderma* and subsequent *C. lindemuthianum* inoculation on seed yield of common bean in the field. (**A**) Anthracnose-free plots: no *C. lindemuthianum* inoculation, (**B**) Anthracnose plots: plants inoculated with *C. lindemuthianum*. Bean cultivars, FJM: Flor de Junio Marcela; FMA: Flor de Mayo Anita; NSL: Negro San Luis; PV: Pinto Villa. Treatments, Control: No *Trichoderma*; B6: *T. asperellum* B6; MK1: *T. longibrachiatum* MK1; P1: *T. atroviride* P1; T22: *T. harzianum* T22. Boxplots indicate medians, 25th and 75th percentiles, whiskers extend to 1.5 times the interquartile range, data points beyond whiskers represent outliers. Asterisks indicate significant effects of *Trichoderma* inoculation on seed yield of plants of the same cultivar and under the same anthracnose-condition (** *p* ≤ 0.01, * *p* ≤ 0.05, ns: *p* > 0.05; based on Wilcoxon post hoc tests of *n* = max. 10 independent biological replicates (or less in cases of high soilborne mortality).

Independently of any statistical considerations, the most important outcome for application purposes is seed yield, at least in the case of grain crops such as common bean. The net effects of resistance induction on plant growth, and ultimately yield, depend on—among other factors—whether the particular disease agent arrives and on the degree to which the specific disease represents a growth- or yield-limiting factor. For example, positive effects of several *T. harzianum* strains and a *T. pseudokoningii* strain on the seedling emergence and early growth rate of pea (*Pisum sativum*) were observed only in *Pythium ultimum*-contaminated soil but not in pathogen-free soil [81]. In our field experiment, *Trichoderma*-inoculation affected seed yield in seven out of 16 strain × cultivar-combinations in the anthracnose-free plots but only three out of 16 strain × cultivar-combinations in the anthracnose-plots (Figure 3).

Kruskal-Wallis tests confirmed significant effects of the cultivar on the seed yield of field-grown plants (Table S4). Most importantly, plants growing in the anthracnose-free plots from *Trichoderma*-inoculated seeds showed significantly lower seed yield than *Trichoderma*-free controls in six individual *Trichoderma* × cultivar-combinations (Figure 3A). Any preventative resistance induction can generate metabolic or other costs which might result in negative effects on seed yield under enemy-free conditions, or when other environmental factors than the particular disease limit seed yield [62]. Evidently, such costs are likely to pay off if they protect the plant from a yield-limiting disease [63,64,65]. Indeed, inoculation of FMA bean seeds with each of the *Trichoderma* strains had a negative effect on seed yield in the anthracnose-free plot, but not so in the presence of anthracnose. In the particular case of T22 inoculation, a significant yield loss of FMA plants on the anthracnose-free plot even converted into a significant increase in yield in presence of the pathogen (Figure 3A,B). However, we also detected a case with opposed effects, i.e., the inoculation of PV beans with T22 resulted in a yield increase under anthracnose-free conditions but not so in the anthracnose-plot (Figure 3A,B).

## 3. Conclusions and Outlook

Carvalho et al. [79] used plastic bags with a sterilized commercial substrate to test the potential of five *T*. *harzianum* strains to favor seedling emergence and subsequent growth from *Sclerotinia sclerotiorum*-infected seeds of common bean (*P. vulgaris*) and obtained results that correlated well with the effects under open field conditions [79], while a study comparing the growth promotion effect of three *T. harzianum* isolates reported opposite effects on root growth for plants cultivated in pots in sterilized field soil versus plants in the open field [82]. In our study, the effects of *Trichoderma* on anthracnose disease of common bean differed depending on the type of soil even when plants were kept in a greenhouse, i.e., under otherwise controlled ambient conditions. In this sense, our results confirm, for common bean, earlier studies that highlighted the soil type as a major determinant of net effects of *Trichoderma* on plant growth or resistance. However, as opposed to our expectation, cultivating beans in field soil in a greenhouse seems not to be an attractive ‘intermediate’ experimental setup, at least if the final aim is the identification of *Trichoderma* strains that reliably reduce anthracnose disease in common bean. We observed the highest levels of disease severity in this specific setup, while plants grown in the open field exhibited the lowest levels. As described earlier for soybean (*Glycine max*) plants infected by *Phakopsora pachyrhizi* [83], the disease levels as observed under greenhouse conditions did not predict the situation in the open field.

Although the diversity of biotic and abiotic factors that varied among our three setups do not allow to relate different results directly to a particular factor, each setup was chosen to represent conditions that are frequently used to evaluate the effects of *Trichoderma* isolates on the growth or disease resistance or—more seldomly—yield of crop plants. Therefore, we consider the lack of reproducibility in the major patterns of anthracnose among the three setups as an important observation. An environment that generates high absolute levels of disease favors the detection of statistically significant effects, but these might be of minor relevance for open field conditions. In fact, with a few exceptions, the results obtained in our greenhouse experiments had little predictive value for the effects under open field conditions. For example, the bean cultivar Negro San Luis (NSL)—a highly susceptible landrace—exhibited the most consistent disease reductions in response to *Trichoderma*, but this disease reduction did not convert into a positive effect on yield. By contrast, we observed no strong effects of inoculation of FMA beans with any of the tested *Trichoderma* strains on anthracnose severity in any of the setups, and *Trichoderma* treatments even were associated with a significant reduction of seed yield of FMA plants growing under anthracnose-free field conditions. However, in presence of the pathogen this cost paid off and in the specific case of T22, a two-fold reduction of seed yield in the absence of anthracnose converted into the opposite effect (a two-fold increase) when plants were challenged with *C. lindemuthianum*. This pattern would be fully congruent with costs of a preventative resistance induction that pay off in presence of the pathogen [84]. However, comparing seed yield reached by each bean genotype on the *C. lindemuthianum* challenged vs. control plots indicates that anthracnose was likely not a major yield-limiting factor in our specific field experiments, a conclusion supported by the lack of a significant effect of anthracnose on seed yield (Table S4). Visual inspection of the plants indicates that seldom but deleterious herbivory (Figure S4) was an important factor of mortality. Although we lack a mechanistic explanation for our observation, our field study has been performed in an area of México where many smallholder farmers cultivate these common bean cultivars, during the same months and usually without any pesticides. Therefore, we consider our work as a case study that calls for stronger emphasis on field experiments and their inclusion already the early phases of screenings.

## 4. Materials and Methods

### 4.1. Biological Material

The seeds of the four common bean cultivars Flor de Junio Marcela, Flor de Mayo Anita, Pinto Villa and Negro San Luis were kindly donated by Dr. Jorge Acosta at the national germplasm collection of Instituto Nacional de Investigaciones Forestales Agrícolas y Pecuarias (INIFAP), Celaya, GTO, México; the *Trichoderma* strains used in this work, *T. longibrachiatum* MK1, *T. asperellum* B6, *T. atroviride* P1 and *T. harzianum* T22, were provided by co-author Dr. Michelina Ruocco from the Institute for Sustainable Plant Protection of the Italian National Research Council (CNR-IPSP, Portici, Naples, Italy), and the *C.* *lindemuthianum* (Sacc. & Magnus) Briosi & Cavara strain 1088 was donated by Dr. June Simpson at CINVESTAV Unidad Irapuato, México.

### 4.2. Culturing Conditions and Inoculation

All fungal strains were cultured on potato dextrose agar (PDA; Difco™, Difco Laboratories, Becton, Dickinson and Company, Sparks, MD, USA), *C. lindemuthianum* in the dark at room temperature and the *Trichoderma* strains in constant light at 28 °C. To prepare suspensions of *Trichoderma* spores for seed inoculation and suspensions of *C. lindemuthianum* conidia for subsequent challenge of bean plants, 10 mL of sterile water with 0.1% Tween (Sigma, St. Louis, MO, USA) were poured over the mycelia to remove spores/conidia and their concentration was adjusted by counting spores/conidia in aliquots in a Neubauer hemacytometer (Hausser Scientific, Horsham, PA, USA).

All bean seeds were surface-sterilized using an ethanol solution (70% *v*/*v*) for 1 min, followed by a sodium hypochlorite solution (5% *v*/*v*) for 5 min, and finally washed three times with sterile distilled water. Seed inoculation with *Trichoderma* followed published protocols [51]. In short, each five seeds were submerged in 3 mL of a spore suspension (1 × 10^7^ spores ml^−1^ with starch at 2% *w*/*v* as adjuvant) and dried overnight in the air flow of a laminar flow hood. Subsequently, seeds were either sown individually in 1.5 L pots (greenhouse setups) or directly into the soil of the field plots. Substrates used in the two greenhouse setups were (1) an autoclaved greenhouse mix consisting of one part loam, two parts mulch, one part vermiculite (SunGro Horticulture, Bellevue, WA, USA), one part perlite (Termolita S.A., Nuevo León, Mexico) and three parts Sunshine Mix 3™ (SunGro Horticulture), and (2) untreated soil collected from the experimental field at CINVESTAV Unidad Irapuato. Conditions in the greenhouse were natural light and photoperiod; average day-time temperature, 28 °C; night-time temperature, 20 °C.

The challenge with *Colletotrichum* was performed in all three setups in the early vegetative phase, i.e., using plantlets with 3–5 trifoliate leaves. The suspension of *C. lindemuthianum conidia* was prepared as described above, adjusted to 1 × 10^6^ conidia ml^−1^ and sprayed directly on both surfaces of the leaves (making sure that both surfaces were completely covered), while control plants were sprayed with sterile water with 0.1% Tween.

### 4.3. Quantification of Disease Severity

The severity of anthracnose disease was quantified 20 days after the challenge with *C. lindemuthianum*. One leaf per plant (*n* = 10 plants per strain × cultivar combination) was randomly selected and scanned using a printer equipped with scanning function (Brother DCP-1602). The total leaf area and the diseased leaf area were quantified using the image analysis software Image J [85] to calculate the percentage of affected area for each leaf. A subset of randomly selected leaves was assigned to confirm infection with *C. lindemuthianum*. Directly after scanning, homogenates prepared from these leaves were plated on solid Potato Dextrose Agar (PDA) (Difco™, Difco Laboratories, Becton, Dickinson and Company, Sparks, MD, USA) to verify the identity *C. lindemuthianum* as re-isolated pathogen based on optical characteristics of the colonies formed (see ref. [69] for details).

### 4.4. Field Experiment: Damage by Non-Target Enemies and Yield

The field experiment was performed in the experimental field of CINVESTAV Unidad Irapuato (State of Guanajuato, 1.800 m above sea level: 20°43′13″ N; 101°19′43″ W) during the spring-summer season of 2019 (from March to August). We established four subplots, two subplots assigned to inoculation with Colletotrichum, the other two subplots as anthracnose-free controls. On 26th and 27th of March, *n* = 8 seeds per strain × cultivar combination and *subplot* were sown directly into the soil in a completely randomized spatial distribution (Figure S2). The emerging plantlets were counted to calculate soilborne mortality rates as the difference from the eight seeds sown per strain × cultivar combination. At the 3–5 leaf stage, plantlets in the two ‘anthracnose plots’ were challenged with *C. lindemuthianum* and leaves were scanned 20 d later as described above. In addition to quantifying the diseased area, the area removed or visibly damaged by herbivores was quantified to calculate herbivore damage as percentage of the total area. Subsequently, plants were allowed to finish their growing cycle. At the end of the reproductive phase, i.e., when plants started to dry naturally, we first counted the number of plants that had survived until reproduction. Plants were harvested individually (either all plants or in case of survival of more than five plants per subplot, a maximum of five randomly selected plants per strain × cultivar combination and subplot), the pods were removed and opened manually and the seeds dried in an oven at 50 °C for two days, to determine seed dry weight per plant.

### 4.5. Data Analysis

All data were analyzed using R version 4.0.2 [86] and R Studio version 1.3.959 [87]. First, global data from the three different experimental sets were analyzed using multivariate analysis of variance (MANOVA). Then, data from each experimental set performed under greenhouse conditions were analyzed individually using non-parametric Kruskal-Wallis test and Wilcoxon post hoc comparison test. Data from greenhouse and field experiments were analyzed using the non-parametric tests mentioned above. Within the same bean genotype, differences between *Trichoderma* treatments were tested for significance using Wilcoxon Pairwise comparison against control group. Graphics were generated using ggplot2 [88] and the R package ggpubr version 0.4.0 [89].

## Figures and Tables

**Table 1:** Results of a Multifactorial Analysis of Variance (MANOVA) for the effects of Setup, Cultivar and *Trichoderma* spp. on anthracnose severity (% diseased leaf area).

Source	df	Sum of Squares	Mean Square	S	*p*
Setup	2	599	299.56	3.7590	0.0238868
Cultivar	3	376	125.33	1.5727	0.1949059
Trichoderma	4	1582	395.51	4.9631	0.0006132
Setup:Cultivar	6	5340	889.93	11.1674	8.470 × 10^−12^
Setup:Trichoderma	8	2430	303.76	3.8118	0.0002237
Cultivar:Trichoderma	12	3447	287.21	3.6041	3.270 × 10^−5^
Setup:Cultivar:Trichoderma	24	7732	322.18	4.0428	8.799 × 10^−10^
Residuals	569	45,344	49.69		

‘Setup’ refers to the experimental setup (Greenhouse: sterile substrate; Greenhouse: field soil; or open field), ‘Cultivar’ refers to the four common bean cultivars, and ‘*Trichoderma*’ refers to the inoculation with one of the four tested *Trichoderma* strains (or no seed inoculation with *Trichoderma*).

**Table 2:** Mortality under field conditions.

Soilborne Mortality											
Anthracnose-Free						Anthracnose Plot					
	Ctrl.	B6	MK1	P1	T22		Ctrl.	B6	MK1	P1	T22
FJM	7	8	7	10	10	FJM	8	8	7	10	11
FMA	4	5	3	6	3	FMA	6	6	4	4	4
NSL	6	5	7	5	8	NSL	5	10	10	11	10
PV	6	10	10	11	8	PV	6	10	8	10	10
**Mortality before Reproduction**											
**Anthracnose-Free**						**Anthracnose Plot**					
	**Ctrl.**	**B6**	**MK1**	**P1**	**T22**		**Ctrl.**	**B6**	**MK1**	**P1**	**T22**
FJM	1	0	4	0	1	FJM	0	1	1	0	0
FMA	0	0	0	0	0	FMA	0	0	0	0	0
NSL	7	9	3	10	6	NSL	9	6	6	3	5
PV	6	0	2	0	0	PV	0	3	0	0	2

Soilborne mortality was determined as not emerged plantlets from 8 seeds sown per strain × cultivar combination in each of the two subplots per anthracnose-condition. Mortality before reproduction was determined as plants that were inoculated with *C. lindemuthianum* but did not reach maturity (i.e., that could be used to quantified yield). Plants in the Anthracnose-plots were inoculated with *C. lindemuthianum*, those in the Anthracnose-free subplots were mock-inoculated with water. Bean cultivars: FJM: Flor de Junio Marcela; FMA: Flor de Mayo Anita; NSL: Negro San Luis; PV: Pinto Villa. Treatments, Ctrl. no *Trichoderma*; B6: *T. asperellum* B6; MK1: *T. longibrachiatum* MK1; P1: *T. atroviride* P1; T22: *T. harzianum* T22.

## Data Availability

The original data of this study are available as a Dryad Dataset, https://doi.org/10.5061/dryad.69p8cz92t.

## Supplementary Materials

The following are available online at https://www.mdpi.com/article/10.3390/plants10081739/s1, Figure S1: Results of Shapiro-Wilk test of normality distribution based on MANOVA test residuals, Table S1: Results of individual Kruskal-Wallis tests of the effects of experimental setup, bean cultivar and *Trichoderma* spp. on anthracnose severity, Table S2: Results of individual Kruskal-Wallis tests of the effects of soil type, bean cultivar and *Trichoderma* spp. on anthracnose severity on plants growing in the greenhouse, Figure S2: Split-plot design and spatial distribution of strain ×cultivar combinations within each plot, Figure S3: Disease symptoms and herbivore-inflicted damage in the field, Table S3: Results of individual Kruskal-Wallis tests of the effects of inoculation with *Colletotrichum*, bean cultivar and *Trichoderma* spp. on herbivore damage (% of removed leaf area) in the field experiment, Figure S4: Damage by chewing herbivores as a major cause of plant mortality, Table S4 Results of individual Kruskal-Wallis tests of the effects of inoculation with *Colletotrichum*, bean cultivar and *Trichoderma* spp. on seed yield (grams per plant) in the field experiment.
