# Supplementary material for: Context-Dependent Effects of Trichoderma Seed Inoculation on Anthracnose Disease and Seed Yield of Bean (Phaseolus vulgaris): Ambient Conditions Override Cultivar-Specific Differences
Source: Plants (Basel). 2021 Aug 23;10(8):1739. doi: 10.3390/plants10081739 (PMC8400414; doi:10.3390/plants10081739)
Supplement: Supplementary file 1 [file plants-10-01739-s001.zip › plants-1309280-supplementary.pdf]

Figure S1. Results of Shapiro-Wilk test of normality distribution based on MANOVA test residuals.

Data	W	*P*
**MANOVA residuals**	0.69399	< 2.2e-16

MANOVA (Multivariate ANOVA) residuals: Df = 569; Sum of Squares = 45344; Mean square = 49.69.

Histogram of MANOVA residuals

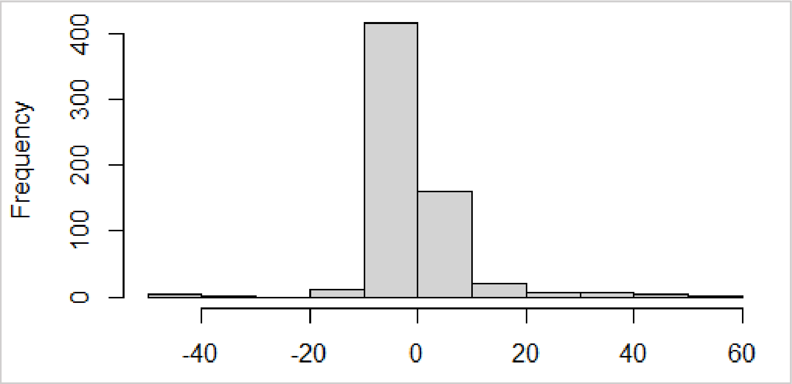

Table S1. Results of individual Kruskal-Wallis tests of the effects of experimental setup, bean cultivar and *Trichoderma* spp. on anthracnose severity.

Factor	Chi-squared	DF	*P*
**Setup**	58.37	2	2.11×10^{-13}
**Cultivar**	9.15	3	0.02742
Trichoderma	19.80	4	0.00055

'Setup' refers to the experimental setup (A. greenhouse: sterile commercial substrate, B. greenhouse: field soil, and C. open field)
'Cultivar' refers to the four bean cultivars tested
'*Trichoderma*' refers to the inoculation of bean seeds with one of the four tested *Trichoderma* strains or no *Trichoderma* inoculation.

Table S2. Results of individual Kruskal-Wallis tests of the effects of soil type, bean cultivar and *Trichoderma* spp. on anthracnose severity on plants growing in the greenhouse.

Factor	Chi-squared	DF	*P*
**Soil type**	49.33	1	2.16×10^{-12}
**Cultivar**	10.26	3	0.01647
Trichoderma	70.10	9	1.46×10^{-11}

The two 'soil types' were sterile commercial substrate and soil collected from the open field
'Cultivar' refers to the four bean cultivars tested
'*Trichoderma*' refers to the inoculation of bean seeds with one of the four tested *Trichoderma* strains or no *Trichoderma* inoculation.

Figure S2. Split-plot design and spatial distribution of strain × cultivar combinations within each plot

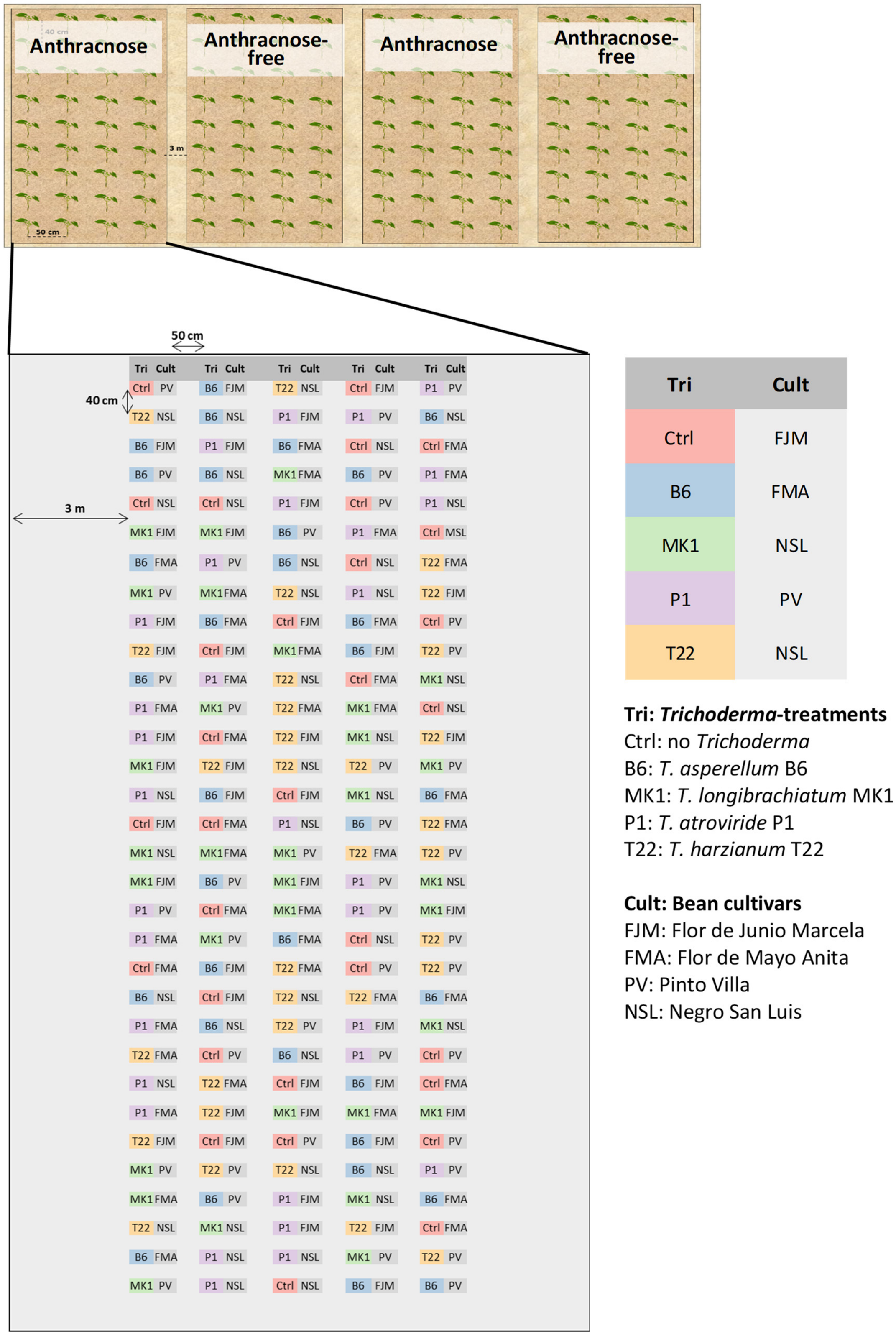

Tri	Cult
Ctrl	FJM
B6	FMA
MK1	NSL
P1	PV
T22	NSL

Tri: *Trichoderma*-treatments
Ctrl: no *Trichoderma*
B6: *T. asperellum* B6
MK1: *T. longibrachiatum* MK1
P1: *T. atroviride* P1
T22: *T. harzianum* T22

Cult: Bean cultivars
FJM: Flor de Junio Marcela
FMA: Flor de Mayo Anita
PV: Pinto Villa
NSL: Negro San Luis

Figure S3 Disease symptoms and herbivore-inflicted damage in the field

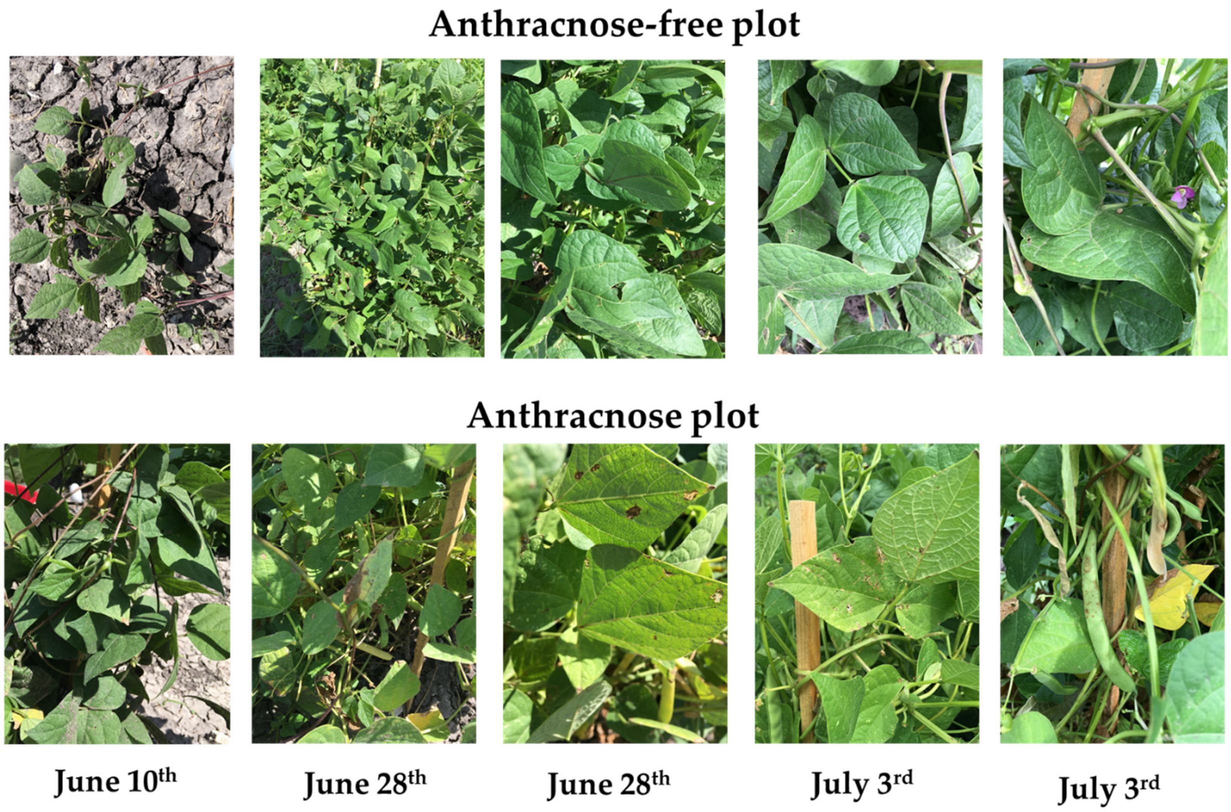

Table S3. Results of individual Kruskal-Wallis tests of the effects of inoculation with *Colletotrichum*, bean cultivar and *Trichoderma* spp. on herbivore damage (% of removed leaf area) in the field experiment.

Factor	Chi-squared	DF	*P*
Colletotrichum	8.99	1	0.0027
**Cultivar**	7.67	3	0.0532
Trichoderma	9.16	4	0.0572

'*Colletotrichum*' refers to the experimental conditions (anthracnose-free plots vs. anthracnose plots),
'Cultivar' refers to the four bean cultivars tested
'*Trichoderma*' refers to the inoculation of bean seeds with one of the four tested *Trichoderma* strains or no *Trichoderma* inoculation.

Figure S4. Damage of young bean plantlets by chewing herbivores as a major cause of plant mortality

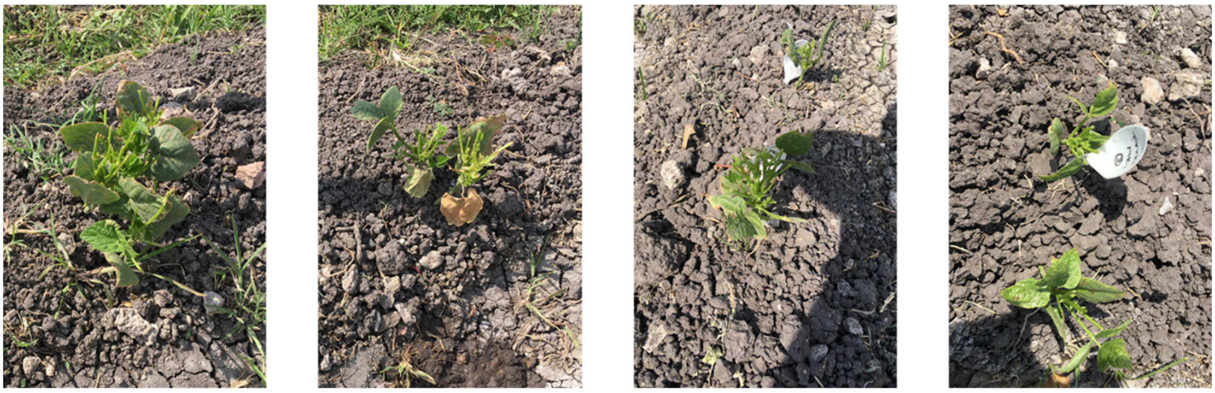

Table S4. Results of individual Kruskal-Wallis tests of the effects of inoculation with *Colletotrichum*, bean cultivar and *Trichoderma* spp. on seed yield (gram seeds per plant) in the field experiment.

**Factor**	**Chi-squared**	**DF**	***P***
Colletotrichum	1.05	1	0.3048
**Cultivar**	201.88	3	2.2×10^{-16}
Trichoderma	6.87	4	0.1429

'*Colletotrichum*' refers to the experimental conditions (anthracnose-free plots vs. anthracnose plots),
'Cultivar' refers to the four bean cultivars tested
'*Trichoderma*' refers to the inoculation with one of the four tested *Trichoderma* strains or no *Trichoderma* inoculation.
